# Non-contrast CT radiomics-clinical machine learning model for futile recanalization after endovascular treatment in anterior circulation acute ischemic stroke

**DOI:** 10.1186/s12880-024-01365-7

**Published:** 2024-07-19

**Authors:** Tao Sun, Hai-yun Yu, Chun-hua Zhan, Han-long Guo, Mu-yun Luo

**Affiliations:** 1 First Clinical Medical College, Gannan Medical University, Ganzhou, Jiangxi China; 2 Department of Medical Ultrasonics, The Third Affiliated Hospital of Gannan Medical University, Ganzhou, Jiangxi China; 3 Department of Neurosurgery, The First Affiliated Hospital of Gannan Medical University, Ganzhou, Jiangxi China

**Keywords:** Anterior circulation Acute Ischemic Stroke, Machine learning, Futile recanalization, Endovascular treatment, Radiomics

## Abstract

**Objective:**

To establish a machine learning model based on radiomics and clinical features derived from non-contrast CT to predict futile recanalization (FR) in patients with anterior circulation acute ischemic stroke (AIS) undergoing endovascular treatment.

**Methods:**

A retrospective analysis was conducted on 174 patients who underwent endovascular treatment for acute anterior circulation ischemic stroke between January 2020 and December 2023. FR was defined as successful recanalization but poor prognosis at 90 days (modified Rankin Scale, mRS 4–6). Radiomic features were extracted from non-contrast CT and selected using the least absolute shrinkage and selection operator (LASSO) regression method. Logistic regression (LR) model was used to build models based on radiomic and clinical features. A radiomics-clinical nomogram model was developed, and the predictive performance of the models was evaluated using area under the curve (AUC), accuracy, sensitivity, and specificity.

**Results:**

A total of 174 patients were included. 2016 radiomic features were extracted from non-contrast CT, and 9 features were selected to build the radiomics model. Univariate and stepwise multivariate analyses identified admission NIHSS score, hemorrhagic transformation, NLR, and admission blood glucose as independent factors for building the clinical model. The AUC of the radiomics-clinical nomogram model in the training and testing cohorts were 0.860 (95%CI 0.801–0.919) and 0.775 (95%CI 0.605–0.945), respectively.

**Conclusion:**

The radiomics-clinical nomogram model based on non-contrast CT demonstrated satisfactory performance in predicting futile recanalization in patients with anterior circulation acute ischemic stroke.

**Supplementary Information:**

The online version contains supplementary material available at 10.1186/s12880-024-01365-7.

## Introduction

Acute ischemic stroke is associated with high morbidity and disability rates [1]. The emergence of endovascular thrombectomy (EVT) as an effective treatment for acute ischemic stroke has been supported by large randomized controlled trials (RCTs) and subsequent meta-analyses [2, 3]. Studies have shown that EVT within 6–24 h can lead to favorable clinical outcomes [4], even in patients with large stroke cores, without significantly increasing the risk of death [5, 6]. Recent reports indicate potential benefits of EVT beyond 24 h as well [7]. Despite successful recanalization (mTICI ≥ 2b), some patients still experience poor outcomes at 90 days (mRS 4–6), which is termed as futile recanalization [8]. Research has highlighted that while a high percentage of patients undergoing EVT achieve successful recanalization, about half of them do not have good 90-day functional outcomes [2, 4]. Therefore, early and accurate prediction of patient outcomes can assist physicians in assessing the patient’s condition, understanding treatment risks and expectations, and devising personalized treatment plans.

With the rise of precision medicine and advancements in scientific technology, machine learning-based radiomics has garnered increasing interest in recent years. This approach shows promising potential in predicting disease outcomes and tumor differentiation, aiding physicians in accurately assessing conditions and providing improved treatment for patients [9].

Radiomics enables the extraction of a large number of quantitative features from medical images, such as shape, intensity, and texture, in an objective and high-throughput manner. Machine learning, on the other hand, effectively manages the relationships between vast amounts of variable data, thereby transforming subjective visual interpretations by physicians into image-driven objective evaluations [10].

Some recent studies have successfully utilized magnetic resonance imaging (MRI) to develop machine learning models for prediction, yielding positive results [11–13]. However, the time-consuming nature of MRI examinations and potential patient cooperation issues have led us to focus on non-contrast CT (NCCT) in this study. NCCT can be obtained quickly and conveniently, aligning with the preoperative examination recommendations outlined by the American Heart Association/American Stroke Association guidelines for acute ischemic stroke [14]. Previous research has highlighted the feasibility of NCCT in predicting recanalization in patients undergoing intravenous thrombolysis [15]. The objective of this study is to leverage machine learning techniques, integrating preoperative imaging radiomics and clinical features, to develop and validate effective predictive models.

## Materials and methods

### Patients and data acquisition

This study was approved by the Ethics Committee and waived informed consent from the patients (LLSC-2023-412).

**Fig. 1:**
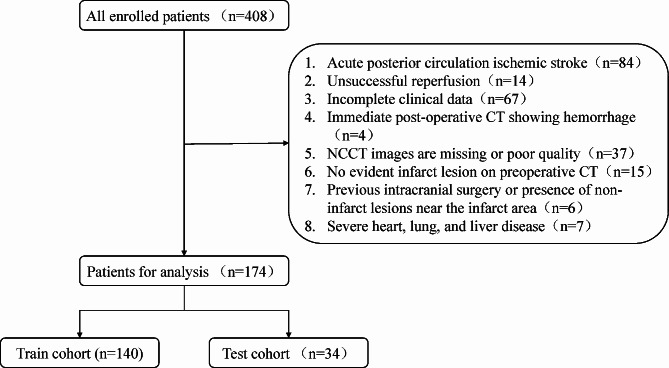
The flow chart for the exclusion of patients

A retrospective data collection was conducted on 174 patients who underwent endovascular treatment for acute anterior circulation ischemic stroke between January 2020 and December 2023. The inclusion criteria were as follows: (1) hospitalization and treatment within 24 h of symptom onset; (2) diagnosis of acute anterior circulation ischemic stroke; (3) receipt of EVT treatment, including mechanical thrombectomy, intra-arterial thrombolysis, balloon angioplasty, stent placement, etc., with successful reperfusion achieved (mTICI score of 2b-3); (4) preoperative NCCT examination. Exclusion criteria were: (1) incomplete patient data; (2) immediate postoperative CT indicating hemorrhage; (3) poor CT image quality with significant artifacts; (4) absence of evident infarct lesion on preoperative CT; (5) history of intracranial surgical treatment or non-infarct lesion in proximity to infarct area; (6) presence of severe cardiovascular, pulmonary, or hepatic diseases. Finally, a total of 174 patients were included. The patients were randomly divided into training cohort (*n* = 140) and testing cohort (*n* = 34) at a ratio of 8:2 (Fig. 1).

Futile recanalization was defined as successful reperfusion after EVT (mTICI ≥ 2b) but poor prognosis at 90 days (mRS 4–6).

Clinical data of the patients were obtained, including clinical text data (such as age, gender, time to vessel reperfusion, site of vascular occlusion, postoperative hyperdensity, hypertension, diabetes, smoking, alcohol consumption, admission blood pressure, etc.) and laboratory data at admission (such as Glu, D-D, FIB, eGFR, WBC, LYMP, MONO, NEUT, etc.). These data were extracted separately from electronic medical records.

All patients who underwent head NCCT examination were examined using GE Discovery CT (GE Medical, Piscataway, NJ, USA) or Somatom Definition Flash CT (Siemens Medical Solutions, Germany). The scanning range was from the top of the head to the base of the skull, with the following scanning parameters: tube voltage 120 kV, tube current 250 mA, slice thickness and interval 5 mm. The NCCT images of all patients were saved in DICOM format.

### Image preprocessing and lesion segmentation

**Fig. 2:**
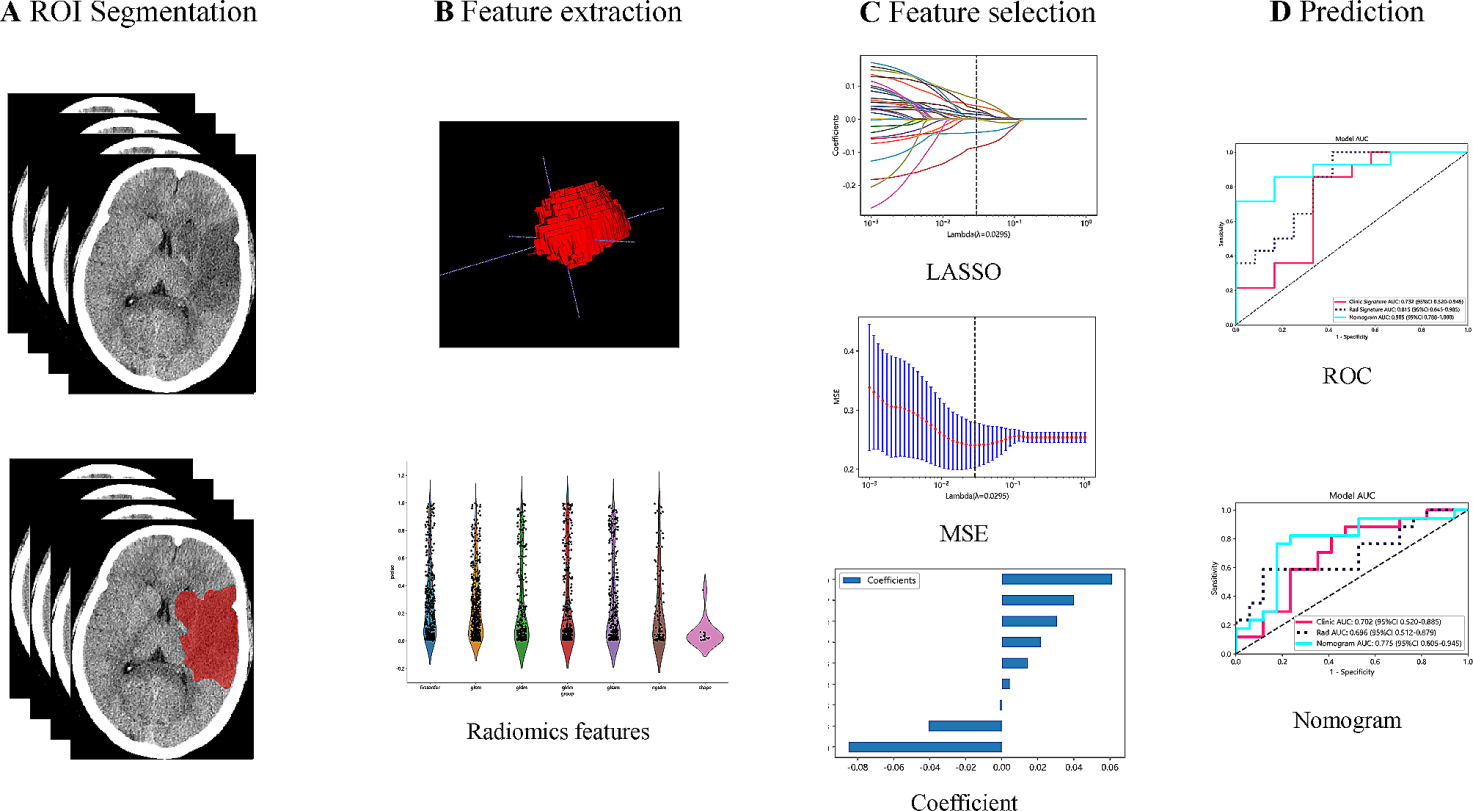
The workflow of the radiomics model construction. **A** ROI segmentation; **B** radiomics features extraction; **C** radiomics feature screening with LASSO; and **D** model building. ROI, regions of interest; LASSO, Least Absolute Shrinkage and Selection Operator; MSE, mean squared error; ROC, receiver operating characteristic curve

Each NCCT image was resampled to achieve a uniform voxel size of 1.0*1.0*1.0 mm. Furthermore, NCCT images were adjusted with a fixed head window (window level = 35 Hounsfield unit (Hu); window width = 60 Hu) to standardize the impact of different instruments and ensure consistent delineation of lesion areas. The main goal of image segmentation was to identify cerebral infarction lesions. The region of interest (ROI) was outlined by an experienced physician (with over 10 years of experience) using ITK-SNAP (Version 3.8.0). The principle of ROI delineation is as follows: the infarct area is annotated layer by layer, with the size of the delineation depending on the size of the infarct focus. The final result is a 3D ROI. In principle, only clearly visible lesions are delineated, as shown in Fig. 2A for the specific delineation effect. In order to ensure the reliability of lesion segmentation and minimize subjective differences, the physician re-outlined the extracted images of 30 patients one month later. The intraclass correlation coefficient (ICC) was employed to assess these features, and only features with ICC ≥ 0.75 were retained.

### Feature extraction

Handcrafted features were extracted using an in-house feature analysis program implemented in Pyradiomics (http://pyradiomics.readthedocs.io) [16]. These features can be categorized into three types: (1) first-order features, (2) shape features, and (3) texture features. First-order features describe the distribution of voxel intensities within the ROI. Shape features describe the geometric characteristics of the ROI in both 2D and 3D. Texture features characterize patterns or spatial distributions of intensities through methods like gray-level co-occurrence matrix (GLCM), gray-level run length matrix (GLRLM), gray-level size zone matrix (GLSZM), and neighborhood gray-tone difference matrix (NGTDM). A total of 2016 radiomic features were extracted from the ROIs, and subsequently standardized using Z-score normalization.

### Feature selection

The study initially conducted Student’s t-test statistical analysis and ICC feature selection on all radiomic features of the training set, retaining only those with a *p*-value < 0.05. For highly correlated features, the Pearson correlation coefficient was calculated, and features with a correlation coefficient of 0.9 or higher were pruned, keeping the feature with the highest absolute correlation to eliminate redundancy. Subsequently, the Max-Relevance and Min-Redundancy (mRMR) method was utilized to further select the 30 most relevant features with minimal inter-feature redundancy. Following this, the Least Absolute Shrinkage and Selection Operator (LASSO) was employed for feature selection, where regression coefficients were shrunk to zero by adjusting the weight parameter λ. The optimal λ was determined using 10-fold cross-validation to minimize the cross-validation error. Features with non-zero coefficients were retained for regression model fitting and combined to create a radiomic signature. Radiomic scores for each patient were then calculated from the linear combination of retained features. After LASSO feature selection, the final selected features were input into a Logistic Regression (LR) model for model construction, with 5-fold cross-validation utilized to obtain the final radiomic signature.

### Clinical model and radiomics-clinical nomogram model construction

The process of building the clinical model is similar to that of the radiomics model. Firstly, features with *p*-value < 0.05 were selected through baseline statistics for model construction. The same machine learning models were used in the construction of the clinical signature. Here, we also employed 5-fold cross-validation to obtain the final clinical model. To visualize the classification evaluation, logistic regression analysis was used to construct a nomogram based on radiomics signature and clinically significant features. Fig. 2 shows the whole process of model building.

### Statistical analysis

Independent t-tests were used for analyzing normally distributed data, while Mann-Whitney U tests were utilized for non-normally distributed data. Chi-square tests were employed for analyzing categorical variables. Receiver operating characteristic (ROC) curves were plotted, and the area under curve (AUC) was calculated to assess the predictive ability of the model. Delong test was used to compare the AUC among three models. Statistical analyses were performed using SPSS (version 21.0; IBM Corporation) and R software (version 4.3.1). A *p*-value < 0.05 was considered statistically significant.

## Results

### Patient characteristics

Initially, 408 patients who underwent EVT surgery were identified, and after screening, 174 patients were finally included. Patients were randomly assigned to training and testing groups. FR patients accounted for 51.4% (72/140) in the training group and 50.0% (17/34) in the testing group. Baseline characteristics of all patients are shown in Table 1.

**Table 1:** Baseline characteristics of patients in cohorts

Characteristic	Training Cohort (*n* = 140, %)	Testing Cohort (*n* = 35, %)	*p*-value
Age	61.73 ± 11.53	61.35 ± 11.94	0.866
Sex			1.000
Male	39(27.86)	10(29.41)	
Female	101(72.14)	24(70.59)	
Hypertension	73(52.14)	15(44.12)	0.517
Diabetes	25(17.86)	5(14.71)	0.855
Smoking	42(30.00)	13(38.24)	0.471
Alcohol drinking	29(20.71)	7(20.59)	1.000
Coronary atherosclerotic heart disease	10(7.14)	2(5.88)	1.000
Atrial fibrillation	13(9.29)	4(11.76)	0.909
IVT	34(24.29)	8(23.53)	1.000
SBP	143.88 ± 26.95	134.59 ± 23.05	0.066
DBP	85.24 ± 17.10	81.15 ± 14.25	0.198
Admission NIHSS	15.22 ± 6.80	14.74 ± 6.24	0.646
Time from symptom onest to reperfusion	10.93 ± 5.08	10.06 ± 5.12	0.340
PCHD	106(75.71)	26(76.47)	1.000
Hyperdense artery sign	74(52.86)	20(58.82)	0.664
Location of arterial occlusion			0.115
ICA	51(36.43)	19(55.88)	
M1	84(60.00)	14(41.18)	
M2	5(3.57)	1(2.94)	
Hemorrhagic Transformation			0.682
No	85(60.71)	18(52.94)	
HI1	7(5.00)	1(2.94)	
HI2	28(20.00)	7(20.59)	
PH1	8(5.71)	4(11.76)	
PH2	12(8.57)	4(11.76)	
Preoperative mTICI			0.719
0	136(97.14)	34(100.00)	
1	4(2.86)		
Postoperative mTICI			0.801
2b	30(21.43)	6(17.65)	
3	110(78.57)	28(82.35)	
D_D	3.21 ± 6.56	5.79 ± 21.53	0.606
FIB	2.71 ± 1.06	2.57 ± 1.16	0.441
Glu	7.84 ± 2.35	7.78 ± 2.05	0.867
UA	347.45 ± 105.72	335.91 ± 113.44	0.772
UREA	5.41 ± 2.57	4.71 ± 2.03	0.213
eGFR	122.55 ± 29.28	133.18 ± 29.20	0.059
RBC	7.35 ± 34.11	5.91 ± 8.50	0.949
WBC	11.23 ± 12.06	9.37 ± 2.68	0.505
LYMP	1.40 ± 0.68	1.39 ± 0.84	0.582
MONO	0.54 ± 0.25	0.65 ± 0.83	0.738
NEUT	8.29 ± 4.12	9.59 ± 13.33	0.689
NLR	8.11 ± 6.92	10.04 ± 16.68	0.857
LMR	2.93 ± 1.50	2.87 ± 1.67	0.599
SIRI	4.47 ± 5.10	18.34 ± 86.01	0.977

Categorical variables are represented by the number (percent), and continuous variables are represented by mean (± standard deviation). IVT, Intravenous Thrombolysis; SBP, systolic blood pressure; DBP, diastolic blood pressure; NIHSS, National Institute of Health Stroke Scale; PCHD, postinterventional cerebral hyper-density; ICA, internal carotid artery; M1, Middle cerebral artery M1 segment; M2, Middle cerebral artery M2 segment; HI, hemorrhagic infarction; PH, parenchymal hemorrhage; mTICI, modified Thrombolysis in Cerebral Infarction; D-D, D-Dimer; FIB, Fibrinogen; Glu, Glucose; UA, Uric acid; UREA, Urea; eGFR, Estimated glomerular filtration rate; RBC, Red Blood Cell; WBC, White blood cell; LYMP, Lymphocyte; MONO, Monocyte; NEUT, Neutrophil; NLR, Neutrophil-to-lymphocyte ratio; LMR, Lymphocyte to monocyte ratio; SIRI, Systemic inflammatory response index

### Radiomics feature selection and model construction

**Fig. 3:**
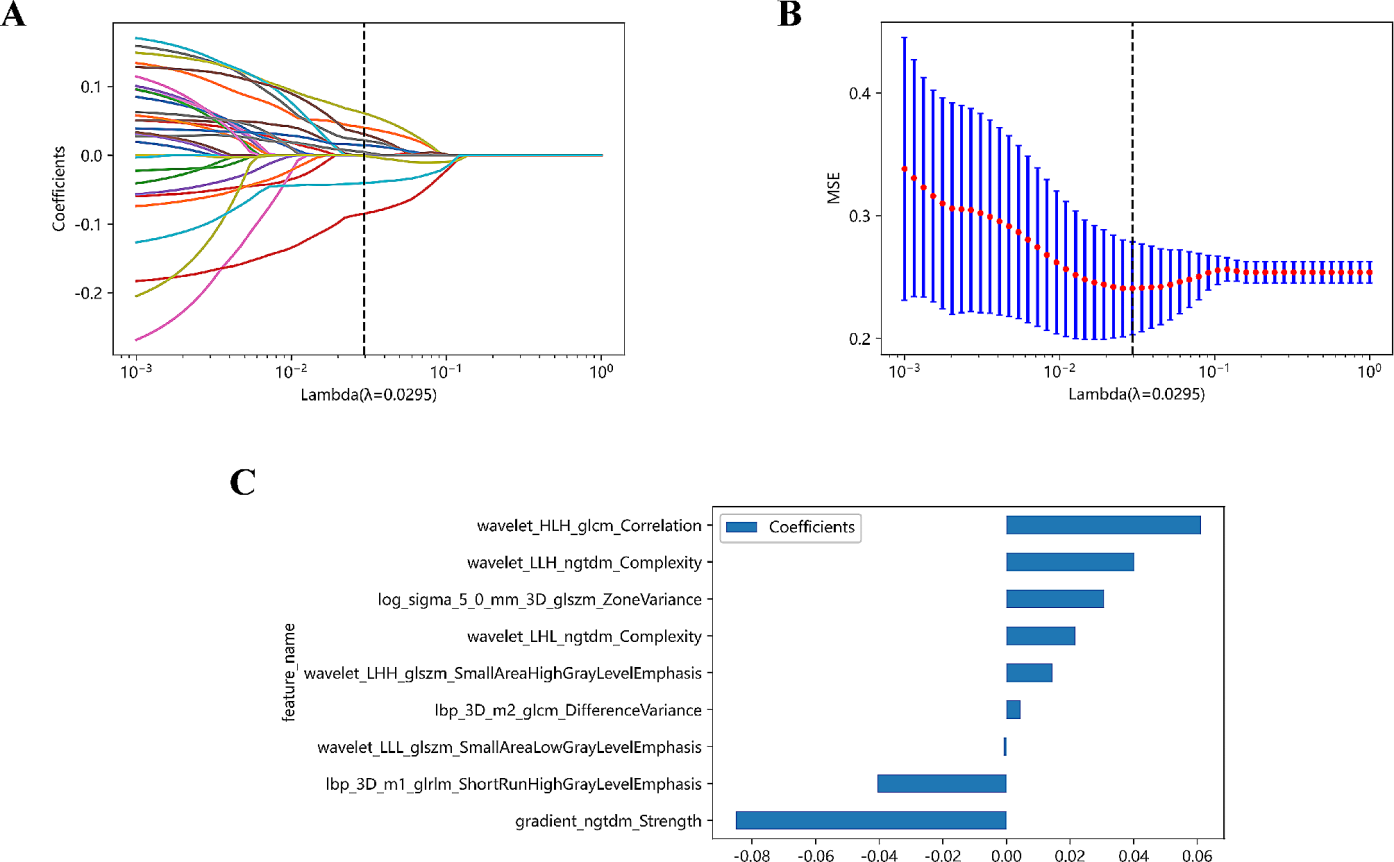
Figures of logistic LASSO regression. (**A**), Lasso path plot of the model in the training dataset. (**B**), Cross-validation plot for the penalty term. (**C**), Pearson correlation coefficients between features were calculated, and 16 features with correlations were retained

**Fig. 4:**
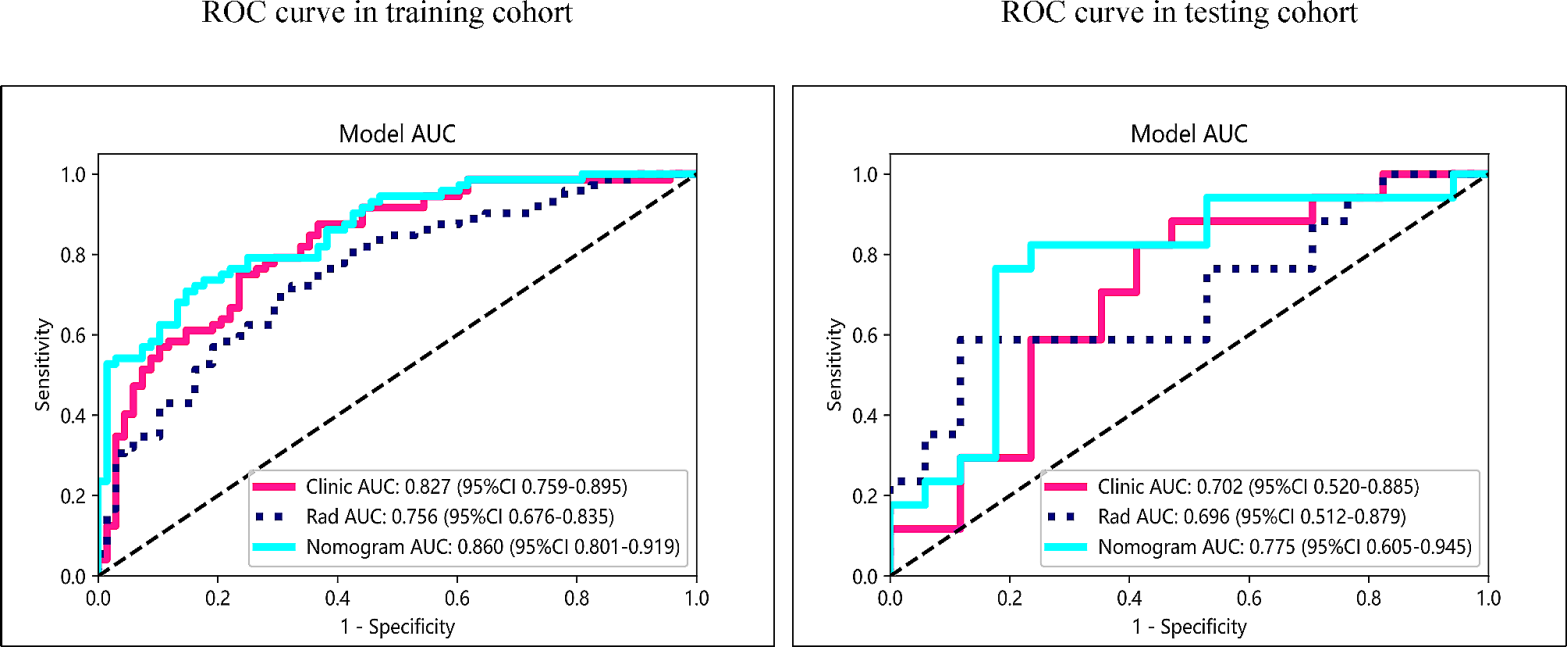
ROC curves of the radiomics model, clinical model, and radiomics-clinical nomogram in the training and test cohort. ROC, receiver operating characteristic

Based on the ROI in patient imaging, a total of 2016 features were extracted for each patient. These features included 396 first-order features, 14 shape features, and 1606 texture features. After conducting ICC and Student’s t-test analyses, 710 stable radiomics features with inter-group differences were identified in the training set. Subsequently, Pearson correlation coefficients were calculated among these features, resulting in the retention of 85 features. Using mRMR method, 30 features with maximum relevance and minimal inter-feature redundancy were selected. Finally, the LASSO method was applied in the training set to determine the optimal regularization weight (λ = 0.0295), resulting in the selection of 9 radiomics features for model construction. Detailed information about these features can be found in Fig. 3. The titles of the nine important features and their corresponding non-zero coefficients are provided in Supplementary Table 1. These features were then input into a Logistic Regression (LR) model for radiomics model construction. The model achieved the AUC of 0.756 (95% CI 0.676–0.835) in the training set, with a sensitivity of 0.681 and specificity of 0.706. In the testing set, the AUC was 0.696 (95% CI 0.512–0.9879), with a sensitivity of 0.529 and specificity of 0.882 (refer to Table 2; Fig. 4 for more details).

**Table 2:** Predictive performance of three models in the Training Cohort and Test Cohort

Model	Training Cohort				Testing Cohort			
	AUC (95%CI)	Accuracy	Sensitivity	Specificity	AUC (95%CI)	Accuracy	Sensitivity	Specificity
Clinic model	0.827 (0.759–0.895)	0.75	0.736	0.765	0.702 (0.520–0.885)	0.676	0.765	0.588
Rad model	0.756 (0.676–0.835)	0.693	0.681	0.706	0.696 (0.512–0.879)	0.706	0.529	0.882
Nomogram	0.86 (0.801–0.919)	0.771	0.694	0.853	0.775 (0.605–0.945)	0.765	0.706	0.824

AUC, area under the receiver operating characteristic curve; CI, confidence interval

### Clinical model and radiomics-clinical nomogram establishment and performance

**Fig. 5:**
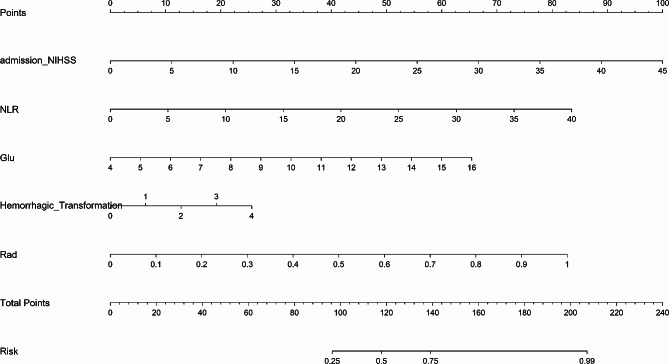
Radiomics-clinical nomogram based on the combined model. Rad_Sig = Radiomics Signature

The clinical model’s features were chosen based on the *p*-value of the training set features (*p*-value < 0.05). Multifactor analysis revealed that admission NIHSS, Hemorrhagic Transformation (HT), Neutrophil-to-Lymphocyte Ratio (NLR), and admission Glu were independent clinical predictive factors (*p*-value < 0.05) (Tables 3 and 4). The clinical model had an AUC of 0.827 (98% CI 0.759–0.895), with sensitivity and specificity of 0.736 and 0.765, respectively, in the training set. In the testing set, the AUC was 0.702 (95% CI 0.520–0.885), with sensitivity and specificity of 0.765 and 0.588, respectively (Table 2; Fig. 4). By combining the radiomics score and clinical predictive factors, the final radiomics-clinical nomogram was developed (Fig. 5). The AUC in the training set and testing set were 0.860 (95% CI 0.801–0.919) and 0.775 (95% CI 0.605–0.945), respectively. The accuracy, specificity, sensitivity, and other metrics of the three models are also detailed in Table 2. The Delong test was performed to compare the AUC of the three models. In the training set, there was a statistically significant difference between the radiomics-clinical nomogram and the Radiomics model (*p* = 0.004), while there was no significant statistical difference between the radiomics-clinical nomogram and the Clinical model (*p* = 0.066), but there was a trend of difference. In the testing set, there was no significant difference between the radiomics-clinical nomogram and the Clinical model (*p* = 0.207) or between the radiomics-clinical nomogram and the Radiomics model (*p* = 0.346) (Supplementary Table 2).

**Table 3:** Univariate analysis for FR in the training cohort

Characteristic	Univariate analysis		
	Meaningful Recanalization (*n* = 68, %)	Futile Recanalization (*n* = 72, %)	*p*-value
Age	60.06 ± 12.29	63.31 ± 10.61	0.096
Sex			0.834
Male	20(29.41)	19(26.39)	
Female	48(70.59)	53(73.61)	
Hypertension	33(48.53)	40(55.56)	0.508
Diabetes	7(10.29)	18(25.00)	0.040
Smoking	26(38.24)	16(22.22)	0.060
Alcohol drinking	18(26.47)	11(15.28)	0.154
Coronary atherosclerotic heart disease	6(8.82)	4(5.56)	0.673
Atrial fibrillation	5(7.35)	8(11.11)	0.635
IVT	15(22.06)	19(26.39)	0.689
SBP	142.75 ± 22.02	144.94 ± 31.01	0.632
DBP	85.69 ± 16.98	84.82 ± 17.32	0.764
Admission NIHSS	12.74 ± 5.51	17.57 ± 7.09	< 0.001
Time from symptom onest to reperfusion	10.94 ± 4.99	10.92 ± 5.20	0.838
PCHD	47(69.12)	59(81.94)	0.116
Hyperdense artery sign	32(47.06)	42(58.33)	0.244
Location of arterial occlusion			0.349
ICA	21(30.88)	30(41.67)	
M1	45(66.18)	39(54.17)	
M2	2(2.94)	3(4.17)	
Hemorrhagic Transformation			0.006
No	49(72.06)	36(50.00)	
HI1	1(1.47)	6(8.33)	
HI2	14(20.59)	14(19.44)	
PH1	3(4.41)	5(6.94)	
PH2	1(1.47)	11(15.28)	
Preoperative mTICI			1.000
0	66(97.06)	70(97.22)	
1	2(2.94)	2(2.78)	
Postoperative mTICI			0.393
2b	12(17.65)	18(25.00)	
3	56(82.35)	54(75.00)	
D_D	2.31 ± 4.15	4.06 ± 8.16	0.001
FIB	2.67 ± 0.91	2.76 ± 1.19	0.874
Glu	7.16 ± 1.99	8.48 ± 2.50	< 0.001
UA	336.78 ± 90.68	357.53 ± 117.95	0.346
UREA	4.78 ± 1.69	6.00 ± 3.09	0.009
eGFR	125.71 ± 28.17	119.57 ± 30.17	0.216
RBC	10.39 ± 48.94	4.48 ± 0.85	0.945
WBC	9.01 ± 3.33	13.33 ± 16.29	< 0.001
LYMP	1.51 ± 0.69	1.30 ± 0.67	0.058
MONO	0.49 ± 0.19	0.59 ± 0.29	0.102
NEUT	6.94 ± 3.16	9.57 ± 4.51	< 0.001
NLR	5.93 ± 5.08	10.16 ± 7.79	< 0.001
LMR	3.24 ± 1.24	2.64 ± 1.66	0.001
SIRI	2.88 ± 3.17	5.97 ± 6.05	< 0.001

Categorical variables are represented by the number (percent), and continuous variables are represented by mean (± standard deviation). IVT, Intravenous Thrombolysis; SBP, systolic blood pressure; DBP, diastolic blood pressure; NIHSS, National Institute of Health Stroke Scale; PCHD, postinterventional cerebral hyper-density; ICA, internal carotid artery; M1, Middle cerebral artery M1 segment; M2, Middle cerebral artery M2 segment; HI, hemorrhagic infarction; PH, parenchymal hemorrhage; mTICI, modified Thrombolysis in Cerebral Infarction; D-D, D-Dimer; FIB, Fibrinogen; UA, Uric acid; UREA, Urea; eGFR, Estimated glomerular filtration rate; RBC, Red Blood Cell; WBC, White blood cell; LYMP, Lymphocyte; MONO, Monocyte; NEUT, Neutrophil; NLR, Neutrophil-to-lymphocyte ratio; LMR, Lymphocyte to monocyte ratio; SIRI, Systemic inflammatory response index

**Table 4:** Multivariate analysis for FR in the training cohort

Characteristic	OR (95%CI)	*p*-value
Smoking	0.917 (0.801–1.05)	0.29
LMR	1.018 (0.957–1.084)	0.631
WBC	1.002 (0.996–1.008)	0.607
NLR	1.033 (1.006–1.061)	0.041
Admission NIHSS	1.024 (1.015–1.035)	< 0.001
SIRI	0.986 (0.954–1.018)	0.467
NEUT	0.984 (0.957–1.012)	0.343
UREA	1.011 (0.985–1.039)	0.474
Glu	1.051 (1.023–1.08)	0.003
Hemorrhagic Transformation	1.064 (1.015–1.114)	0.03
Diabetes	0.965 (0.807–1.154)	0.741
MONO	1.889 (1.099–3.248)	0.054

NIHSS, National Institute of Health Stroke Scale; Glu, Glucose; UREA, Urea; WBC, White blood cell; MONO, Monocyte; NEUT, Neutrophil; NLR, Neutrophil-to-lymphocyte ratio; LMR, Lymphocyte to monocyte ratio; SIRI, Systemic inflammatory response index

## Discussion

Acute ischemic stroke is a global health concern associated with high morbidity and disability rates [1]. Recent large RCTs and meta-analyses have shown that EVT and thrombolytic therapy are effective treatments for acute ischemic stroke [2, 3]. Despite successful reperfusion, a significant number of patients still have poor outcomes at 90 days, termed futile recanalization, affecting almost half of treated patients [2, 4, 8]. Early prediction of patient outcomes can help physicians understand the patient’s condition, assess treatment risks and expectations, and personalize treatment plans.

Zhou et al. developed an imaging-genomic model using DWI and ADC, along with clinical indicators, to predict the prognosis of acute anterior circulation ischemic stroke [12]. Their model achieved high AUCs in both training and test cohorts, outperforming single clinical models. Similarly, Luo et al. used DWI to predict the prognosis of posterior circulation ischemic stroke and demonstrated the superiority of the clinical-radiomics model over clinical models [17]. These studies emphasize the importance of integrating imaging, genomic, and clinical data to enhance the accuracy of predicting patient outcomes in acute ischemic stroke. By utilizing advanced technologies like radiomics, clinicians can make more informed decisions and provide personalized care for stroke patients, ultimately improving treatment efficacy and patient outcomes. MRI examinations are time-consuming, potentially hindering patient cooperation and impacting recanalization in patients. NCCT was chosen as the focus of this study due to its faster and more convenient acquisition, in line with AIS guidelines [14].

A model was developed in this study to predict FR by combining clinical data with NCCT features based on radiomics. This approach improves diagnostic accuracy by providing specific quantitative indicators, reducing misdiagnosis and missed diagnosis due to lack of physician experience. The study identified four independent predictors of FR outcomes upon admission: NIHSS, hemorrhagic transformation, NLR, and admission blood glucose. NIHSS scores, commonly used to assess stroke severity, were found to be a key indicator for evaluating acute ischemic stroke outcomes, consistent with previous studies [18].

Research has shown that neuroinflammation is a key factor in both the development and advancement of acute ischemic stroke [19, 20]. When cerebral tissue experiences ischemia, the release of harmful substances from damaged cells, including inflammatory cytokines and chemokines, can lead to the breakdown of the blood-brain barrier (BBB). This breakdown allows immune-inflammatory cells to enter the brain, contributing to secondary brain injury.

Neutrophils (NEUT) are one of the earliest blood-derived cell populations to enter the brain following an acute ischemic stroke (AIS) and are a significant component of thrombi in AIS patients [21]. They play a role in disrupting the blood-brain barrier, limiting neoangiogenesis and repair, promoting neuronal death, among other effects, by producing matrix metalloproteinase-9 (MMP-9) and neutrophil extracellular traps (NETs) [22, 23]. On the other hand, lymphocytes are thought to have a neuroprotective function [24]. The NLR is a biomarker that reflects the balance between neutrophils and lymphocytes, providing insight into baseline inflammation and immune status [25]. Studies have indicated that a high NLR upon admission can predict functional outcomes at discharge in patients undergoing intravenous thrombolysis. Higher NLR values are linked to poorer short-term functional outcomes in AIS patients and may potentially prolong hospital stays, aligning with our own research findings [26, 27].

Additionally, elevated levels of blood glucose upon admission have been associated with increased BBB disruption, leading to worse outcomes and more symptomatic intracranial hemorrhages [28]. The impact of high blood glucose on the microcirculation exacerbates ischemic injury and blood-brain barrier damage. Mechanisms through which high blood glucose contributes to HT may involve oxidative stress and inflammation [29]. Research by Research conducted by Desilles et al. supports the idea that high blood glucose triggers a thrombo-inflammatory cascade, intensifying downstream microvascular thrombo-inflammation due to cerebral artery occlusion, worsening reperfusion injury, and ultimately leading to BBB disruption and HT occurrence [30, 31].

HT refers to brain hemorrhage occurring within the area of primary ischemic stroke. As per the European Cooperative Acute Stroke Study standards, HT can be radiologically classified as hemorrhagic infarction (HI) and parenchymal hematoma (PH) [32]. Recent research indicates that HI-2, PH-1, and PH-2 are independent predictive factors for poor prognosis in AIS patients following successful EVT [33].

Additionally, changes in monocyte counts have been observed. Ischemic-hypoxic stimulation in cerebral infarction prompts monocytes to produce inflammatory mediators like interleukin-1 (IL-1), IL-6, IL-8, and tumor necrosis factor (TNF), leading to excessive inflammation that worsens brain tissue damage [34]. Consequently, there is a proposal for using monocyte count as a predictor of stroke outcomes [35].

The Delong test in this study only revealed statistical significance between the radiomics-clinical nomogram and the radiomics model in the training cohort. However, it is important to highlight that the AUC of the radiomics-clinical nomogram consistently outperformed both the single clinical model and the radiomics model, indicating that the fusion model, with its integration of more features, demonstrates superior predictive performance compared to individual models. This underscores the potential of radiomics in predicting FR.

Despite the intriguing findings of our study, it is important to acknowledge several limitations. Firstly, there is a potential for selection bias due to the exclusion of patients with incomplete data. Additionally, being a single-center retrospective study, the lack of patients from other medical centers hinders the generalizability of the findings, and the relatively small sample size limits the practical application of the model. As a result, further validation through large-scale prospective randomized controlled trials is necessary. Furthermore, the study did not utilize more advanced techniques like deep learning and automatic image segmentation. Future research endeavors will incorporate deep learning methods in the next phase.

In summary, the radiomics-clinical machine learning model based on NCCT demonstrates superior accuracy in predicting FR in AIS patients compared to standalone clinical or radiomics models. This has the potential to assist clinicians in developing personalized treatment plans for patients early in the disease course, ultimately improving the prognosis of stroke patients.

## Conclusion

The radiology-clinical machine learning model, utilizing preoperative NCCT data, demonstrated promising results in predicting futile recanalization in patients with anterior circulation ischemic stroke. This model has the potential to assist neurologists in evaluating patient prognostic outcomes promptly, offering valuable insights for personalized treatment strategies.

### Electronic supplementary material

Below is the link to the electronic supplementary material.

Supplementary Material 1

## Data Availability

The data that support the findings of this study are not openly available due to reasons of sensitivity and are available from the corresponding author upon reasonable request.

## Abbreviations

FR: Futile recanalization
AIS: Acute ischemic stroke
LASSO: Least absolute shrinkage and selection operator
LR: Logistic regression
AUC: Area under the curve
EVT: Endovascular thrombectomy
RCTs: Randomized controlled trials
MRI: Magnetic resonance imaging
NCCT: Non-contrast CT
ROI: Region of interest
ICC: Intraclass correlation coefficient
ROC: Receiver operating characteristic
mRMR: Max-Relevance and Min-Redundancy
NLR: Neutrophil-to-Lymphocyte Ratio
BBB: Blood-brain barrier
