# Supplementary material for: Non-contrast CT radiomics-clinical machine learning model for futile recanalization after endovascular treatment in anterior circulation acute ischemic stroke
Source: BMC Med Imaging. 2024 Jul 19;24:178. doi: 10.1186/s12880-024-01365-7 (PMC11264869; doi:10.1186/s12880-024-01365-7)
Supplement: Supplementary file 1 — Supplementary Material 1 [file 12880_2024_1365_MOESM1_ESM.docx]

**Supplementary table 1** Nine radiomics features screened by Lasso regression and their corresponding nonzero coefficients.

| Radiomics feature | Coefficient |
| --- | --- |
| wavelet_LLH_ngtdm_Complexity | 0.040102 |
| wavelet_LHL_ngtdm_Complexity | 0.021666 |
| wavelet_HLH_glcm_Correlation | 0.061109 |
| log_sigma_5_0_mm_3D_glszm_ZoneVariance | 0.030718 |
| lbp_3D_m2_glcm_DifferenceVariance | 0.004440 |
| wavelet_LHH_glszm_SmallAreaHighGrayLevelEmphasis | 0.014450 |
| gradient_ngtdm_Strength | -0.085030 |
| wavelet_LLL_glszm_SmallAreaLowGrayLevelEmphasis | -0.000940 |
| lbp_3D_m1_glrlm_ShortRunHighGrayLevelEmphasis | -0.040550 |

**Supplementary table 2** Delong test

|  | Nomogram Vs Clinic | Nomogram Vs Rad |
| --- | --- | --- |
| Training Cohort | 0.066 | 0.004 |
| Testing Cohort | 0.207 | 0.346 |
